# Implementing hospital guidelines improves warfarin use in non-valvular atrial fibrillation: a before-after study

**DOI:** 10.1186/1471-2458-7-203

**Published:** 2007-08-10

**Authors:** Simona Bo, Susanna Valpreda, Luca Scaglione, Daniela Boscolo, Marina Piobbici, Mario Bo, Giovannino Ciccone

**Affiliations:** 1 Department of Internal Medicine, University of Torino, Italy; 2 Unit of Cancer Epidemiology, San Giovanni Battista Hospital, Torino, Italy; 3 Department of Geriatrics, University of Torino, Italy

## Abstract

**Background:**

The use of oral anticoagulant therapy (OAT) to prevent non-valvular atrial fibrillation (NVAF) related-strokes is often sub-optimal. We aimed to evaluate whether implementing guidelines on antithrombotic therapy (AT) by a multifaceted strategy may improve appropriateness of its prescription in NVAF-patients discharged from a large tertiary-care hospital.

**Methods:**

A survey was conducted on all consecutive NVAF patients discharged before (1^st ^January–30^th ^June 2000, *n *= 313) and after (1^st ^January–30^th ^June 2004, *n *= 388) guideline development and implementation.

**Results:**

When strongly recommended, OAT use increased from 56.6% (60/106 in 2000) to 81.9% (86/105 in 2004), with an absolute difference of +25.3% (95%CI: 15% 35%). In patients for whom the choice OAT/acetylsalicylic acid should be individualised, those discharged without any AT were 33.7% (34/101) in 2000 and 16.9% (21/124) in 2004 (-16.7%;95%CI: -26.2% -7.2%). In a logistic regression model, OAT prescription in 2004 was increased by 2.11 times (95%CI: 1.47 3.04), after accounting for stroke risk, presence of contraindications (OR = 0.18; 0.13 0.27), older age (OR = 0.30; 0.21 0.45), prophylaxis at admission (OR = 3.03; 2.08 4.43). OAT was positively associated with the stroke risk in the 2004 sample only.

**Conclusion:**

The guideline implementation has substantially improved the appropriateness of OAT at discharge, through a better evaluation at patient's individual level of the benefit-to-risk ratio.

## Background

Non-valvular atrial fibrillation (NVAF) is associated with a six-fold increased stroke risk, accounting roughly for 20–25% of ischaemic stroke in older patients [1]. With the increasing population ageing, the burden of NVAF is expected to double in the near future, with important public health implications [2]. It has been shown that oral anticoagulation therapy (OAT) and acetylsalicylic acid (ASA) can respectively reduce the risk of stroke by 50–60% and 10–15% in patients at higher thromboembolic risk (stroke rate >6%/year), while the absolute benefit is much less evident for lower risk patients (stroke rate ≤2%/year) [3]. Further, the effectiveness of OAT is well demonstrated in clinical practice, without an exceeding risk of bleeding complications, even in older patients [4,5]. Nevertheless, recent surveys have shown that warfarin is still largely underused, being the proportion of treated patients lower than 50% among those for whom OAT would be appropriate [6-8]. Uncertainty about the benefit/risk balance, fear of major bleeding complications and poor compliance to coagulation monitoring are the main factors accounting for under-prescription of OAT by clinicians [5].

We recently conducted an audit on the appropriateness of antithrombotic therapy (AT) at discharge in patients with NVAF in a large tertiary-care teaching hospital in Turin (Northern Italy) [6]. From patients at high/very high stroke risk without OAT contraindications: less than 50% were properly treated with warfarin, about 30% received ASA, but more than 20% were discharged without any AT. As a consequence of these unsatisfactory findings, a locally-adapted guideline for the prevention of cardioembolic events in patients with NVAF was developed and implemented in the years 2002–2003.

In the present paper we report the impact of this guideline on the appropriateness of AT in patients with NVAF, through a before-and-after controlled study.

## Methods

All consecutive patients discharged alive with "chronic NVAF" coded as a secondary diagnosis (International Classification of Disease, code 427.31) were identified from the discharge files of S. Giovanni Battista Hospital of Turin in the period 1^st ^January–30^th ^June 2000, and 1^st ^January–30^th ^June 2004.

Exclusion criteria were: 1) NVAF as main diagnosis, since cardioversion and anticoagulation could be employed according to specific protocols; 2) age >90 years; 3) discharge from surgical units, because of OAT contraindications (eg recent surgery) or complications (eg NVAF as a transient arrhythmia after open-heart surgery). Both permanent and recurrent NVAF (at least two ECG-documented episodes either before entry or during hospitalisation) without potential reversible causes, were considered, since criteria for anticoagulation are the same [9].

The clinical records were abstracted by two authors, using a standardised form. Preliminary random sample of 10% of the records were independently abstracted by both authors; areas of disagreement were discussed.

All patients gave their written informed consent to analysis of data from their clinical records, and all procedures were in accordance with the Declaration of Helsinki.

Risk factors for thromboembolic events were: previous ischaemic stroke/transient ischaemic attack (TIA)/embolic event, age ≥ 65 years, hypertension (previous diagnosis; use of anti-hypertensive drugs), diabetes mellitus (chronic elevation of glycaemia; hypoglycaemic medication use), heart failure (history/diagnosis in the records), documented left ventricular systolic dysfunction (ejection fraction <40%), coronary artery disease (CAD) (current/previous chronic angina pectoris; acute coronary syndrome; coronary re-vascularisation).

The following were considered as OAT contraindications: pro-haemorrhagic/coagulative disorders, intra-cranial haemorrhage (history/current), major bleeding within 6-months (requiring transfusion/hospitalisation), severe impaired renal function, hepatic cirrhosis, severe psychiatric disease, dementia, unreliable patient, severe uncontrolled hypertension, history of recurrent falls (≥ 2), chronic alcoholism, known allergic reaction to warfarin, previous discontinuation of OAT because of bleeding, life-expectancy <12 months, difficulty/refusal by the patient of coagulation monitoring.

A large multidisciplinary panel, composed of 19 physicians from the hospital departments of emergency, internal medicine, geriatrics, cardiology, neurology, haematology, epidemiology, and a general practitioner, worked during 2002–2003 to develop a guideline on AT for NVAF. The guideline of the Scottish Intercollegiate Guidelines Network (SIGN) was adapted (see below). A review of the literature, through electronic databases (Medline, Cochrane Library), was undertaken to search papers worth consideration published after the SIGN guideline and to obtain further data about OAT bleeding risk. The guideline was adopted in June 2003 [10].

On the basis of the available evidence, OAT resulted associated with a greater risk of bleeding in patients ≥ 75 y [1]. A risk stratification model, taking into account the estimated thromboembolic and bleeding risk according to age below and ≥ 75 years, and the newer risk stratifications proposed [11], was developed (Table 1). The use of OAT was considered as either recommended, to be individualised, or not recommended. In the absence of contraindications, OAT was strongly recommended for patients of any age at very high risk, and in <75 y patients at high risk. OAT or ASA were considered appropriate (and the choice to be individualised) in patients without contraindications at moderate stroke risk (of any age), and in high risk ≥ 75 y patients. Since OAT contraindications include conditions with varying degrees of severity, the guideline recommended that in the presence of any contraindication and high-very high thromboembolic risk, the decision about AT should be based on a careful individual evaluation of the risk/benefit balance. Lastly, OAT was considered not recommended in patients at low-moderate stroke risk and with contraindications.

**Table 1:** Guideline risk stratification and recommended antithrombotic therapy.

**Classes of thromboembolic risk:**	**Estimated stroke risk (%/year):**	Patients' characteristics and risk factors*:	**Recommendations†:**
Very high risk	12	Previous ischaemic stroke or TIA or other embolic event	OAT strongly recommended
High risk, age <75	>5	≥65 years and at least one risk factor	OAT strongly recommended
High risk, age ≥75	>5	≥65 years and at least one risk factor	OAT or ASA recommended
Moderate risk	3–5	<65 years and at least one risk factor or ≥65 years and no risk factors	OAT or ASA recommended
Low risk	≤1	<65 years and no risk factors	OAT not recommended

* Risk factors for thromboembolic events include: hypertension, diabetes mellitus, heart failure or left ventricular systolic dysfunction, coronary heart disease. **†**In patients with contraindications to OAT and high or very high risk of stroke the recommended prophylaxis was considered uncertain: any decision about the use of OAT, ASA or no prophylaxis should be individualised, balancing the benefits with the risks due to the contraindications. OAT, oral anticoagulant therapy; ASA, acetylsalicylic acid.

A before-discharge contact with the general practitioner to decide the best way of OAT monitoring was suggested; outpatient monitoring in anticoagulation clinics for elderly or more complicated patients was encouraged.

One of the main task was to identify possible barriers to the guideline adoption. A major issue was the fear of bleeding caused by OAT, particularly in the elderly. This issue was addressed by providing physicians with clear recommendations, including a weighted balance between thromboembolic and haemorrhagic risks.

A report of the results of the initial audit hold in 2000, showing a large under-utilisation of OAT [6] was extensively distributed in the hospital wards. The guideline was presented to all hospital physicians, sample copies and a reminder with an easy-to-use coloured table for AT indications were distributed. Dedicated meetings in each unit were organised by the multidisciplinary group by scheduling sessions. The guideline was published on a local medical journal, distributed to all the family practitioners, and ad hoc meetings for them were organised.

The main and the secondary outcomes of the project were, respectively: an increase in the appropriate OAT prescription at discharge, and the increment in patients discharged with any AT. Since only one patient resulted at low thromboembolic risk, this subject was included in the moderate risk category.

Based on the results of the first audit, we estimated that a new sample (at least 300 patients) would be required to detect with sufficient precision (alpha = 0.05, 2-sided and beta = 0.20) an absolute increase of at least 15% of OAT (from 55% to 70%), when strongly recommended (about 1/3 of the total sample).

The absolute difference between proportions was the main outcome measure; 95% confidence intervals were calculated using Confidence Interval Analysis, version 2.1.1. A logistic regression analysis was performed to evaluate the guideline impact on OAT use at discharge, after adjustments for age, risk of stroke, OAT contraindications, AT at admission; the logistic models were also stratified by period (SAS, version 8.2).

## Results

Clinical characteristics of patients with NVAF (n = 313 in 2000; n = 388 in 2004), are presented in Table 2. Compared to patients discharged in 2000, those evaluated in 2004 were slightly older, had higher prevalence of hypertension, heart failure, and lower prevalence of previous stroke/TIA. The presence of at least one OAT contraindication was recorded in 106/313 patients (33.9%) in 2000 and in 159/388 patients (41.0%) in 2004.

**Table 2:** Clinical characteristics and distribution of patients according to the recommended treatment, by period of discharge.

**Clinical characteristics**	**2000**		**2004**	
	
	**N**	**(%)**	**N**	**(%)**
**Patients**	**313**	**(100.0)**	**388**	**(100.0)**
Males	159	(50.8)	193	(49.7)
< 70 years	65	(20.8)	71	(18.3)
70–79 years	135	(43.1)	163	(42.0)
≥ 80 years	113	(36.1)	154	(39.7)
Hypertension	232	(74.1)	326	(84.0)
Previous stroke/TIA	100	(31.9)	95	(24.5)
Diabetes mellitus	77	(24.6)	91	(23.4)
Heart failure	146	(46.6)	209	(53.9)
Coronary heart disease	96	(30.7)	114	(29.4)

**Recommended treatment:**	**N**	**(%)**	**N**	**(%)**

OAT strongly recommended*:	**106**	**(33.9)**	**105**	**(27.1)**
▪ Very high risk of stroke	62	(19.9)	45	(11.6)
▪ High risk of stroke, age < 75	44	(14.0)	60	(15.5)
**OAT or ASA recommended^†^:**	**101**	**(32.3)**	**124**	**(32.0)**
▪ High risk of stroke, age ≥ 75	67	(21.4)	94	(24.2)
▪ Moderate risk of stroke	34	(10.9)	30	(7.7)
**Uncertain^‡^**	**96**	**(30.7)**	**146**	**(37.6)**
▪ Very high risk of stroke	58	(18.5)	96	(24.7)
▪ High risk of stroke	38	(12.1)	50	(12.9)
**OAT not recommended^§^**	**10**	**(3.2)**	**13**	**(3.3)**

*Patients without contraindications to OAT and a favourable balance for AT, for whom OAT is the preferred choice. †Patients without contraindications to OAT and a favourable balance for AT, but the choice between OAT or ASA should be individualised. ‡Patients with contraindications to OAT and high or very high risk of stroke: any decision about the use of OAT, ASA or no AT should be individualised, balancing the benefits with the risks due to the contraindications. §Patients with contraindications to OAT and low-moderate risk of stroke. OAT, oral anticoagulant therapy; AT, antithrombotic therapy; ASA, acetylsalicylic acid.

The distribution of patients according to the guideline recommendations, by discharge period, is shown in Table 2.

In the second audit, the percentage of patients discharged with OAT, when strongly recommended, increased significantly from 56.6% to 81.9% (absolute increase: +25.3%) (Table 3). When both OAT/ASA were appropriate, the proportion of patients treated with OAT increased from 40.6% to 54.8%. OAT use increased also in patients with an uncertain benefit/risk balance (from 12.5% to 29.5%), but not among patients for whom it was clearly not recommended (Table 3). Overall, the prevalence of patients discharged with any AT (OAT/ASA), when recommended, increased from 158/207 (76.3% in 2000) to 204/229 (89.1% in 2004), with an absolute increase of +12.8% (95%CI:6.8% 18.7%).

**Table 3:** Patients discharged with OAT or with any treatment (OAT/ASA), by the recommended treatment, before-and-after implementation.

	**2000 (N = 313)**					**2004 (N = 388)**					**Absolute difference (2004–2000) in the prevalence of patients discharged with:**			
	
Recommended treatment*:	**Total**	**OAT**		**OAT or ASA**		**Total**	**OAT**		**OAT or ASA**		**OAT**		**OAT or ASA**	
	
	**N**	**N**	**(%)**	**N**	**(%)**	**N**	**N**	**(%)**	**N**	**(%)**	**%**	**(95%CI)**	**%**	**(95%CI)**
**OAT strongly recommended**	106	60	(56.6)	91	(84.8)	105	86	(81.9)	101	(96.2)	+25.3	(14.9 34.9)	+10.3	(3.9 17.2)
**OAT or ASA recommended**	101	41	(40.6)	67	(66.3)	124	68	(54.8)	103	(83.1)	+14.2	(3.2 24.8)	+16.7	(7.2 26.2)
**Uncertain**	96	12	(12.5)	50	(52.1)	146	43	(29.5)	112	(76.7)	+17.0	(8.1 24.9)	+24.6	(14.3 34.5)
**OAT not recommended**	10	2	(20.0)	5	(50.0)	13	1	(7.7)	8	(61.5)	-12.3	(-39.0 12.2)	+11.5	(-20.6 41.1)

*See footnote to table 2

The use of OAT *at admission*, among patients for whom OAT was strongly recommended, increased from 36.8% to 58.1% (+21.3%), while the prevalence of patients either treated with ASA or receiving no AT drugs declined (from respectively 29.2% to 23.8%, and 34.0% to 18.1% -data not shown-). Overall, the percentage of patients without OAT contraindications admitted with AT (OAT or ASA) increased from 58.4% in 2000 to 74.7% in 2004 (*p *< 0.001).

The OAT prescription at discharge was analysed by a logistic regression model, including the following variables: study-period, stroke risk, OAT contraindications, age, AT at admission (Table 4). The adjusted OR for OAT prescription was 2.11 (95%CI:1.47 3.04) for the study period (2004 vs 2000). Age >75 and presence of contraindications were both strong negative predictors of OAT, without variation between periods. Meanwhile, OAT prescription was positively associated with the patient's stroke risk only in the 2004 sample. AT at admission was a much stronger predictor of OAT therapy at discharge in 2000 than in 2004.

**Table 4:** Predictors of OAT prescription at discharge in the whole sample, before, and after the guideline.

	**Whole sample**		**Period:**			
			**2000**		**2004**	
	
	OR*	**(95% CI)**	OR*	**(95% CI)**	OR*	**(95% CI)**
**Risk of stroke:**						
▪ **Moderate**	1	-	1	-	1	-
▪ **High**	0.99	(0.55 – 1.76)	0.45	(0.19 – 1.03)	2.18	(0.96 – 4.94)
▪ **Very high**	1.48	(0.78 – 2.80)	0.58	(0.23 – 1.44)	3.92	(1.55 – 9.93)
**Contraindications to OAT:**						
▪ **None**	1	-	1	-	1	-
▪ **One or more**	0.18	(0.13 – 0.27)	0.17	(0.09 – 0.34)	0.19	(0.11 – 0.30)
**Age:**						
▪ **< 75**	1	-	1	-	1	-
▪ **≥ 75**	0.30	(0.21 – 0.45)	0.35	(0.19 – 0.62)	0.26	(0.15 – 0.43)
**AT at admission:**						
▪ **No**	1	-	1	-	1	-
▪ **Yes**	3.03	(2.08 – 4.43)	4.88	(2.74 – 8.69)	2.07	(1.23 – 3.47)
**Period:**						
▪ **2000**	1	-	-	-	-	-
▪ **2004**	2.11	(1.47 – 3.04)	-	-	-	-

(*)Odds ratios (OR) and 95% confidence intervals (95% CI) adjusted for all the variables listed in the table with logistic regression models.

OAT, oral anticoagulant therapy; AT, antithrombotic therapy.

## Discussion

The implementation of guidelines for AT in NVAF patients may be an effective tool for improving appropriateness of OAT prescription at discharge. An absolute 25% increase in the appropriate prescription of OAT at discharge was observed. Remarkably, very few patients were discharged without any AT in 2004 as compared with 2000. An absolute 14% increase in OAT use in 2004 as compared with 2000 was also observed among patients for whom the decision on best AT should be individualised.

Epidemiological surveys indicated that there is a temporal trend in the use of OAT: the proportion of patients with NVAF treated with warfarin ranged from 20% to 53% in the early 90s [12,13], rose to 48–55% in the years 1995–1997 [14,15], and up to 50–58% around the year 2000 [16,17]. It appears that most of the increasing trend in warfarin use occurred within the period 1992–1994, few years after the publication of the clinical trials between 1989–1992 [14], while in more recent years OAT seems to have reached a plateau [8].

Few studies aimed at improving appropriateness of AT prophylaxis in patients with NVAF have been reported. The combination of physician/nurse education and use of an OAT monitoring service have led to substantial improvements both in the percentage of properly treated patients (88%), and in the incidence of effective anticoagulation among the ambulatory patients [18]. The use of a patient decision aid on AT in NVAF was associated with a 12% absolute improvement in the number of patients receiving appropriate therapy, even if this beneficial effect did not persist [19]. Programs of guideline implementation for general practitioners, or within hospitals, determined a significant increase in warfarin use in high risk patients, even though the prevalence of properly treated patients was still sub-optimal, ranging from 46% to 73% [20,21]. A multifaceted intervention involving nine Australian teaching hospitals resulted in a higher rate of AT prescription (92%), although the authors did not stratify patients according to their thromboembolic risk, but considered appropriate AT use in the absence of contraindications [22]. A pharmacist-led-multidisciplinary intervention in older in-patients increased AT use (from 60% to 81%), even if about a fifth of patients were still discharged without any prophylaxis [23].

Previous studies demonstrated that in clinical practice OAT prescription is often in weak accordance with the individual patients' risk of stroke, and that the non-use of warfarin is not always motivated by the presence of OAT contraindications.

Several clinician-related concerns (including the fear of bleeding complications, the perceived poor compliance of patients to long-term therapy) and poor risk perception together with misunderstanding of the true risk/benefit balance of OAT by patients themselves, might account for OAT under-use even in patients without contraindications to its use [24,25]. Therefore, some studies documented a poor OAT use rather than an under-use: older patients at high risk were often not given warfarin in favour of younger, low-risk AF patients [26]. In order to overcome these potential drawbacks when deciding AT, we developed guidelines including a list of contraindications to OAT and recommendations for AT expressed as age/specific risk/benefit balance. Overall, the proportion of patients with no contraindications who were discharged with OAT doubled in 2004 as compared with 2000. Remarkably, an increase of OAT use was observed only in high or very high risk patients (+17%), for whom the appropriate balance between risks and benefits is uncertain, while it was reduced in moderate risk patients (-12%), where OAT was clearly not recommended.

Even if implementing guidelines may also drive some potential side effects, as previously observed in our hospital, where the higher rates of appropriate procedure use were associated with higher rate of inappropriate use [27], the present findings clearly indicate that the guideline implementation improved the appropriateness of OAT prescription, rather than simply extending its use. Indeed, warfarin use at discharge was strongly associated with the individual stroke risk level only in 2004, while its use was negatively associated with older age and presence of contraindications in both study periods. Thus, a general positive impact of the guideline on improvement of the clinical practice by a better evaluation of the individual benefit-to-risk ratio has been achieved by a tailored implementation strategy of a locally adapted guideline.

More efficient ways to transfer important evidence-based knowledge into usual clinical practice are needed, in order to further reduce the proportion of patients discharged without any prophylaxis (e.g. feedback to physicians, alternative care delivery programs, including increased patient participation) [28].

Our study was conducted in a large teaching hospital: although our data may reflect a local reality, the proportion of properly treated patients is not discordant from previous reports in other countries [20,23].

As in all chart-review based-studies, there is a potential for misclassification bias. Although data were carefully abstracted and jointly evaluated, we cannot exclude that some important information regarding the anticoagulation decision and/or existing contraindications to warfarin, might have been missed.

A common limitation of the before/after design in evaluating the health intervention effectiveness is the possibility that the changes observed are merely expression of a temporal trend. This trend is well documented by the increased proportion of patients on OAT at admission. However, this should not be the case of our findings, because multivariate analysis showed that OAT appropriateness at discharge was improved, as a consequence of the project, also after taking into account AT at admission.

The CHADS2 score, a recently proposed and validated stroke risk index, allows an easy and practical risk stratification [29]. However, at the time the hospital guideline was planned, it was validated in a single study on a large not-European cohort. Only recently, in fact, the predictive role of this score has been validated in an Italian cohort [30].

The classification of the American College of Chest Physician (ACCP) represents another scheme for cardioembolic risk stratification [31]. Our scheme differs from the ACCP classification with regard to the following points: i) a higher weight was attributed to prior stroke/TIA, which by itself determined a very high risk, in line with CHADS2 criteria; ii) other risk factors were given the same weight; iii) the hemorrhagic risk of treatment was combined with its antithrombotic effect, and age ≥ 75 years was considered as a cut-off associated with a greater bleeding risk with OAT use [1].

Recently, the American College of Cardiology, the American Heart Association, and the European Society of Cardiology proposed a joint stratification which recognised a higher risk to previous stroke/TIA or embolism, and gave the same weight to diabetes, hypertension, heart failure/left ventricular dysfunction, which were moderate risk factors [32].

All these schemes did not consider the bleeding risk, when recommending the treatment of choice.

## Conclusion

Our intervention, which involved a large group of end-users in implementing a hospital guideline, has improved the clinical practice through a better evaluation at the patient's individual level of the benefit to risk ratio in deciding the appropriate AT.

The follow-up of these patients will tell us whether this improved clinical practice might be maintained in the long-term, and whether it may translate into improved outcomes.

## Competing interests

The author(s) declare that they have no competing interests.

## Authors' contributions

SB participated in the conception and design of the study, data analysis, interpretation of the findings of the study, manuscript writing and revision.

SV participated in the data collection, interpretation of the findings, manuscript revision.

LS participated in the data collection, interpretation of the findings, manuscript revision.

DB participated in the data collection, interpretation of the findings, manuscript revision.

MP participated in the data collection, manuscript revision.

MB participated in the interpretation of the findings, manuscript writing and revision.

GC participated in the conception and design of the study, data analysis, interpretation of the findings of the study, manuscript writing and revision.

All authors read and approved the final manuscript

## Pre-publication history

The pre-publication history for this paper can be accessed here:


